# Cryptotanshinone Inhibits Bladder Cancer Cell Malignant Progression in a Lipopolysaccharide-Induced Inflammatory Microenvironment through NLRP3 Inhibition

**DOI:** 10.1155/2024/8828367

**Published:** 2024-01-30

**Authors:** Chenye Tang, Xiao Guo, Yu Li, Yongxiang Yi, Zhiling Tang, Qihui Zhang, Bairu Luo, Kean Chen, Ke Liang, Gang Li

**Affiliations:** ^1^Department of Urology, The First Affiliated Hospital of Soochow University, Suzhou 215006, China; ^2^Department of Urology, The Second Hospital of Jiaxing, Jiaxing 314000, China; ^3^The Fourth School of Clinical Medicine, Zhejiang Chinese Medical University, Hangzhou 310053, China; ^4^Department of Clinical Pathology, Jiaxing Master Degree Cultivation Base, Zhejiang Chinese Medical University, Jiaxing 314001, China; ^5^Department of Urology, The First People's Hospital of Pinghu, Jiaxing 314299, China

## Abstract

**Background:**

Bladder cancer (BC) is one of the most common malignancies of the urogenital system. This study assessed the nucleotide-binding oligomerization domain and leucine-rich repeat and pyrin domain-containing protein 3 (NLRP3) in BC as well as the effects of cryptotanshinone on changes in BC malignant behaviors and NLRP3 expression under a lipopolysaccharide (LPS)-induced inflammatory microenvironment.

**Methods:**

BC tissue specimens from 62 patients were collected for immunohistochemical detection of NLRP3 protein. BC and normal urothelial cell lines were cultured for the detection of NLRP3 mRNA and protein. Then, BC cells were pretreated with LPS to mimic the inflammatory tumor microenvironment. Next, these cells were incubated with a low or high dose of cryptotanshinone to assess its effects on tumor cell malignant behaviors as well as transfected with NLRP3 cDNA to confirm the role of NLRP3 in BC cells *in vitro*.

**Results:**

High NLRP3 expression was associated with larger tumor diameters (>2 cm), muscle invasion, and metastasis. The levels of NLRP3 mRNA and protein were greater in BC cells than in normal urothelial cells. LPS pretreatment significantly promoted NLRP3 and inflammatory cytokine expression in BC cells, and induced cell viability, migration, and invasion. However, cryptotanshinone was able to reduce the LPS-induced increase of NLRP3 and inflammatory cytokine expression as well as the BC cell malignant progression. NLRP3 overexpression using NLRP3 cDNA further promoted BC cell malignant progression after LPS stimulation and reversed cryptotanshinone-reduced LPS-induced BC cell malignant behaviors.

**Conclusion:**

NLRP3 might possess oncogenic activity in BC, and the antitumor activity of cryptotanshinone in BC *in vitro* might be related to its inhibition of NLRP3 expression.

## 1. Introduction

Bladder cancer (BC) is a commonly diagnosed urological malignancy, accounting for an estimated 573,000 new cases and 213,000 deaths in 2020 globally [1]. Histologically, BC can be classified into nonmuscle-invasive bladder cancer (NMIBC) and muscle-invasive bladder cancer (MIBC); the majority of BC patients are initially diagnosed as having NMIBC, but tumor recurrence will cause NMIBC to eventually become MIBC [2, 3]. BC treatment depends on the stage of the disease; for example, NMIBC is currently treated with transurethral resection of the bladder tumor with or without intravesical chemotherapy and immunotherapy (e.g., Bacillus Calmette–Guerin therapy), while MIBC is treated with radical cystectomy, neoadjuvant chemotherapy, or transurethral resection plus chemoradiation [3, 4]. After initial successful tumor treatment and control, the 5-year risk of NMIBC recurrence ranges from 30% to 80%, while the 5-year survival of MIBC patients is only approximately 50% [5]. Thus, further investigation of novel treatment options for BC that can better control BC progression and provide a favorable prognosis is urgently needed.

Inflammation, as a protective response of the human body against different bacterial and viral pathogens, damaged cells, or irritants [6, 7], is also considered as an important factor for the development and progression of various human cancers due to its regulation of tumor immunosuppression, angiogenesis, and tumor cell proliferation and metastasis [8]. In other words, the tumor microenvironment, containing numerous infiltrated inflammatory cells, plays a key role in cancer development and progression as the inflammatory tumor microenvironment [9]. Literature reports also indicate that the inflammatory microenvironment contributes to BC angiogenesis and progression [7, 10]. Furthermore, as a component of the Gram-negative bacterial cell wall, lipopolysaccharide (LPS) is an important inflammatory factor that triggers tissue inflammation, thus inducing neuritis, pneumonia, and sepsis [11–13]. LPS is able to bind to the toll-like receptor 4 (TLR4)/myeloid differentiation-2 complex and activate TLR4 signaling to phosphorylate and translocate nuclear factor kappa B to the cell nuclei, in turn promoting the transcription of the nucleotide-binding oligomerization domain, leucine-rich repeat, and pyrin domain-containing protein 3 (NLRP3). This protein is a part of the innate immune system and functions as a pattern recognition receptor to recognize pathogen-associated molecular patterns and activate the NLRP3 inflammasome [14, 15]. LPS can also activate caspase-11 to promote NLRP3 expression [16]. Subsequently, activated NLRP3 can activate caspase-1, which releases interleukin (IL)-1*β* and IL-18 to induce tissue inflammation in the human body [17]. In addition to the inflammatory responses, NLRP3 also has been shown to be associated with the progression of various human cancers, including colorectal and breast cancers as well as acute myeloid leukemia [17–19]. Therefore, NLRP3 has been speculated to be a novel target for tumor therapy [20]. Moreover, cryptotanshinone, a lipophilic diterpenoid quinone extracted from the dried root and rhizome of *Salvia miltiorrhiza* Bunge, has been shown to be a potent anti-inflammatory agent and antitumor agent [21, 22].

In this study, we first detected the NLRP3 level in BC tissue specimens for its association with the clinicopathological features of patients. After that, we assessed the effects of cryptotanshinone on changes in BC malignant behaviors and NLRP3 expression under an LPS-induced inflammatory microenvironment. Upon completion of our proposed experiments, we expect that our findings will provide useful information regarding NLRP3 in BC development and the use of cryptotanshinone as a therapeutic agent for the control of BC progression in the future.

## 2. Materials and Methods

### 2.1. Patients and Tissue Specimens

We collected BC tissue specimens from 62 patients (45 males and 17 females, aged 33–88 years old; mean age: 68.74 ± 10.32 years). These patients were histologically diagnosed with BC and treated with transurethral resection of the bladder tumor, partial cystectomy, or radical cystectomy without any preoperative chemotherapy or immunotherapy between May 2019 and September 2022 at the Second Hospital of Jiaxing (Jiaxing, China). The clinicopathological data, such as the patient's age, sex, body mass index, history of alcohol consumption and tobacco smoking, disease history (e.g., hypertension and diabetes), and current tumor classifications (e.g., tumorigenesis frequency, tumor number, diameter, invasive type, metastasis, and histological grade) derived from their medical records in our hospital electronic medical record system. The paraffin-embedded tissue blocks were obtained from the Department of Pathology and used for immunohistochemical analysis of NLRP3 expression.

### 2.2. Immunohistochemistry

The paraffin-embedded tissue specimens from these 62 BC patients were prepared into 4-*μ*m tissue sections. For immunohistochemistry, these tissue sections were first deparaffinized using xylene, rehydrated using a gradient of alcohol solutions, and then subjected to antigen retrieval by submersion into 10 mM citrate buffer (pH 6.0; HaoKe Biotechnology, Hangzhou, China) in a microwave oven at 98°C for 20 min. After that, the sections were washed briefly in phosphate-buffered saline (PBS; HaoKe Biotechnology) three times and then blocked in 3% H_2_O_2_ in 100% methanol for 25 min to inactivate potential tissue peroxidase activity. Next, the sections were treated with 3% bovine serum albumin (HaoKe Biotechnology) in PBS at room temperature for 30 min and then incubated with a primary antibody against NLRP3 (Proteintech, Rosemont, IL, USA) at 4°C overnight. On the next morning, the sections were first bathed in PBS and then in the horseradish peroxidase (HRP)-conjugated goat antirabbit IgG (Abcam, Cambridge, UK) at room temperature for 50 min. The positive signal on the tissue sections was visualized after submersion in diaminobenzidine (Abcam) solution briefly at room temperature and the positive signal under a microscope showed a brown color. The sections were finally counterstained with hematoxylin (HaoKe Biotechnology) for 2–3 min, then dehydrated, cleared with an increasing gradient of ethanol solutions and xylene, respectively, and mounted using neutral gum. The immunostained tissue sections were reviewed, classified, and photographed under a Nikon microscope (Tokyo, Japan). The NLRP3 expression was categorized into four levels according to the intensity of the immunohistochemical staining (see details in the Results section).

### 2.3. Cell Lines, Culture Conditions, and Treatment

Human BC (5637, J82, T24, and TCCSUP) cells and human urothelial cells immortalized with SV-40 (SV-HUC-1), obtained from the Chinese Academy of Type Culture Collection Cell Bank (Shanghai, China), were cultivated in Roswell Park Memorial Institute medium-1640 (RPMI-1640; Gibco, Gaithersburg, MD, USA) supplemented with 10% fetal bovine serum (FBS; Invitrogen, Carlsbad, CA, USA) at 37°C in a humidified incubator with 5% CO_2_. The BC 5,637 cell line was then treated with LPS (100 *μ*g/mL; Sigma–Aldrich, St. Louis, MO, USA) and/or the selective NLRP3 inhibitor MCC950 (1 *μ*M; MedChemExpress, Monmouth Junction, NJ, USA) for 12 hr and then with cryptotanshinone (2 or 4 *μ*M; Sigma–Aldrich) for an additional 24 hr, while dimethyl sulfoxide (MedChemExpress) was used as a vehicle control. Meanwhile, the plasmid pcDNA3.1-NLRP3 (Jiangsu Genecefe Biotechnology, Wuxi, China) carrying NLRP3 cDNA was added to 5,637 cells with the help of Lipofectamine 2000 (Invitrogen) to induce NLRP3 expression.

### 2.4. Reverse Transcription–Quantitative Polymerase Chain Reaction (RT-qPCR)

Cellular RNA was isolated from cells using TRIzol reagent (Invitrogen) and then reversely transcribed into cDNA using the PrimeScript RT Master Mix (Takara, Shiga, Japan), according to the manufacturer's instructions. These cDNA samples were then amplified using qPCR with the PowerUp SYBR Green Master Mix (Thermo Fisher Scientific, Waltham, MA, USA) in an Applied Biosystems 7500 Real-Time PCR System (Thermo Fisher Scientific), according to the manufacturer's protocol. The qPCR amplification conditions were set to an initial denaturation at 95°C for 2 min and then 40 cycles of 95°C for 15 s, 60°C for 20 s, and 72°C for 30 s. The mRNA expression level of NLRP3 was normalized against GAPDH mRNA (a control), and the relative NLRP3 expression was normalized using the 2^−*ΔΔ*Ct^ method. The human NLRP3 mRNA primers were 5′-GAGGCTGGCATCTGGATGAG-3′ and 5′-CGGGGCTATGACATTGGACA-3′, while the GAPDH primers were 5′-GACAGTCAGCCGCATCTTCT-3′ and 5′-GCGCCCAATACGACCAAATC-3′.

### 2.5. Western Blot

Cellular protein was extracted from cells using radioimmunoprecipitation assay buffer (Invitrogen) and quantified with a bicinchoninic acid protein assay kit (Beyotime Biotechnology, Shanghai, China), according to the manufacturers' protocols. The denatured protein samples were separated in 10% sodium dodecyl sulfate–polyacrylamide gels and transferred onto polyvinylidene fluoride membranes (Millipore, Burlington, MA, USA). For Western blotting, the membrane was first incubated in 5% nonfat milk in Tris-buffered saline (TBS) at room temperature for 1 hr and then with a primary antibody against NLRP3, cyclin D1, cyclin-dependent kinase 4 (CDK4), matrix metalloproteinase (MMP)-2, MMP-9, E-cadherin, vimentin, or GAPDH (Proteintech) at 4°C overnight. After that, the membrane was briefly washed with TBS-Tween-20 (Sigma–Aldrich) three times and then incubated with HRP-labeled goat antirabbit IgG at room temperature for 1 hr. Subsequently, the positive protein signals were visualized using an enhanced chemiluminescence kit (Beyotime Biotechnology). The images were captured by using an automatic gel imaging system (Clinx Science Instruments, Shanghai, China), while GAPDH protein was used as a control.

### 2.6. Enzyme-Linked Immunosorbent Assay (ELISA)

The cell culture supernatant was collected and used to determine the levels of IL-1*β*, IL-18, and tumor necrosis factor-alpha (TNF-*α*). In brief, the collected supernatant was centrifuged at 3,000 *g* for 10 min to remove any cell debris and then subjected to ELISA to detect these inflammatory factors using individual ELISA kits (Beyotime Biotechnology), according to the manufacturer's protocols. The optical density (OD) value was measured at 450 nm by using an automatic microplate reader (Thermo Fisher Scientific) and then analyzed accordingly.

### 2.7. Cell Viability Assay

The cell counting kit-8 (CCK-8; Yeasen Biotechnology, Shanghai, China) was utilized to assess the changed cell viability in BC 5,637 cells after different treatments. In brief, tumor cells at a density of 5 × 10^3^ cells per well were plated into a 96-well plate and treated for up to 96 hr. Thereafter, the CCK-8 reagent was added to the cell culture wells, the cells were continuously cultured for an additional 2 hr at 37°C, and the OD values were detected with a microplate reader at 450 nm. The cell viability was determined using the following formula: Relative cell viability= OD value_450 nm_ in the observation group/mean OD value_450 nm_ in the control group × 100%. The assay was conducted in triplicate and repeated three times.

### 2.8. Tumor Cell Colony Formation Assay

The ability of BC 5,637 cells to form colonies after different treatments was assessed by performing colony formation experiments. In particular, cells were seeded into 6-cm culture dishes containing 1,500 cells per dish and incubated at 37°C for 2 weeks, during which time, the cells were treated with various reagents for different durations (see above for details) and the cell growth medium was replaced every 4 days. Next, the cells were fixed with 100% methanol for 2 min, then stained with crystal violet solution for 15 min, and photographed under an inverted Nikon microscope in five randomly selected high-powered microscopic fields. Finally, the cell colonies (≥50 cells) were counted using ImageJ software (National Institutes of Health, Bethesda, MD, USA).

### 2.9. Transwell Invasion Assay

The invasion capacity of BC 5,637 cells after various treatments was detected using a transwell invasion assay. Specifically, the transwell inserts (Corning, Corning, NY, USA) were first precoated with 50 *μ*L of Matrigel (Corning) at 37°C for 6 hr, 1 × 10^5^ 5,637 cells were inoculated into the upper chamber, RPMI-1640 containing 20% FBS was put into the bottom chamber, and then the cells were cultured for 24 hr. The cells remaining on the top surface of the inserts were removed using a cotton swab, whereas the cells that had invaded into the bottom surface of the inserts were fixed with 70% ethanol and stained with crystal violet solution for 20 min. The cells on the stained inserts were counted under an inverted Nikon microscope at 400x magnification for five randomly selected microscopic fields. The tumor cell invasion level was determined using the following formula:

(1) $$\begin{array}{c} \text{Relative tumor cell invasion rate}=\frac{\text{Number of invaded cells in the observation group}}{\text{Mean number of invaded cells in the control group}}\times 100\%. \end{array}$$

### 2.10. Wound Healing Assay

The migration ability of BC 5,637 cells was assayed using a tumor cell wound healing assay. Briefly, tumor cells were plated into a 24-well cell culture plate at 1 × 10^4^ cells per well and cultured overnight to reach 95%–98% confluency at the cell monolayer. Next, the cells were wounded by using a sterile 200-*µ*L pipette tip for multiple damages, then washed with PBS to remove the cell debris and floating cells, and incubated for an additional 24 hr. The cells were photographed at 0 and 24 hr under an inverted Nikon microscope at 200x magnification, and the wounds were quantified by using ImageJ software. The cell migration ability was analyzed using the following formula:

(2) $$\begin{array}{c} \text{Tumor cell migration rate}=\left(1-\frac{{\text{Wound area}}_{24\text{hr}}}{{\text{Wound area}}_{0\text{hr}}}\right)\times 100\%. \end{array}$$

### 2.11. Statistical Analysis

The experiments in this study were performed with three independent replicates and repeated three times. The data were summarized as the mean ± standard deviation. The data were then statistically analyzed using one-way analysis of variance combined with Tukey's test for multiple group data, while the chi-squared test was performed for the comparison between the clinicopathological data and NLRP3 expression. *P* < 0.05 was set as being statistically significant.

## 3. Results

### 3.1. High NLRP3 Protein Expression Was Associated with Tumor Diameter, Invasive Type, and Metastasis in BC

**Figure 1:**
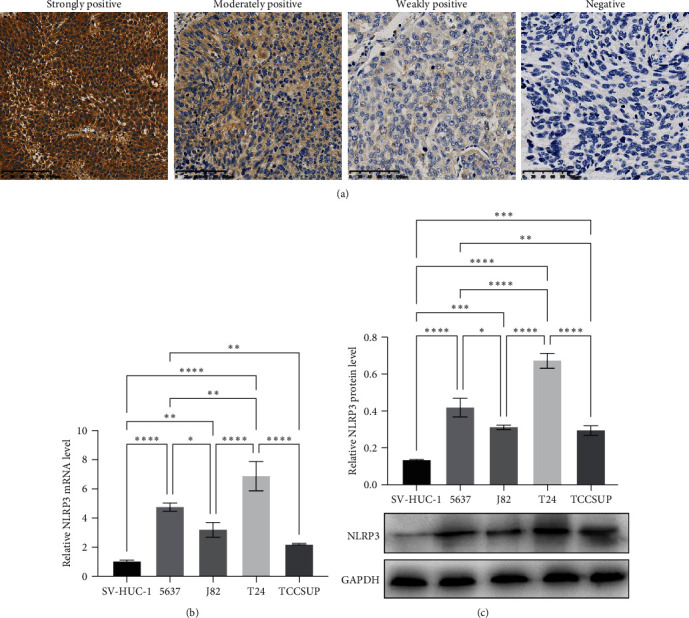
Expression level of NLRP3 in bladder cancer tissues and cells (5637, J82, T24, and TCCSUP) vs. a normal urothelial cell line (SV-HUC-1). (a) NLRP3 protein was detected in bladder cancer tissues using immunohistochemistry (scale bar, 100 *μ*m). Cell lines were grown and subjected to (b) RT-qPCR analysis of NLRP3 mRNA and (c) Western blot analysis of NLRP3 protein.  ^*∗*^*P*  < 0.05,  ^*∗∗*^*P*  < 0.01,  ^*∗∗∗*^*P*  < 0.001, and  ^*∗∗∗∗*^*P*  < 0.0001.

In this study, we first assessed NLRP3 protein expression in BC tissues immunohistochemically. The NLRP3 level in BC tissues was categorized into strongly positive, moderately positive, weakly positive, or negative staining, according to the immunostaining intensity under a microscope (Figure 1(a)). Among the 62 BC patients analyzed, we found 17 strongly positive, 19 moderately positive, 20 weakly positive, and six negative cases of NLRP3 protein expression. The chi-squared test demonstrated that NLRP3 expression was significantly associated with the tumor diameter, invasive type, and metastasis, but there was no association with the patient's sex or age, tumorigenesis frequency, number of tumors, histological grade, tobacco smoking or alcohol consumption history, hypertension, diabetes, or the body mass index. In brief, high rates of strong NLRP3 expression occurred in patients with a tumor diameter >2 cm, MIBC, and tumor metastasis (Table 1).

We then assessed the expression level of NLRP3 in different BC cell lines (5637, J82, T24, and TCCSUP) vs. the normal urothelial cell line SV-HUC-1. RT-qPCR analysis revealed that the mRNA level of NLRP3 was greater in 5,637, J82, and T24 cells compared to that in SV-HUC-1 cells (Figure 1(b)), while Western blot assays showed that NLRP3 protein was more highly expressed in all four BC cell lines compared to that of SV-HUC-1 cells (Figure 1(c)). These four cell lines were ranked from high to low expression of NLRP3 as follows: T24, 5637, J82, and TCCSUP (Figures 1(b) and 1(c)). Therefore, we selected 5,637 cells with a moderate level of NLRP3 expression for the subsequent experiments.

### 3.2. LPS Upregulated the Levels of NLRP3 and Inflammatory Cytokine Expression in BC Cells

**Figure 2:**
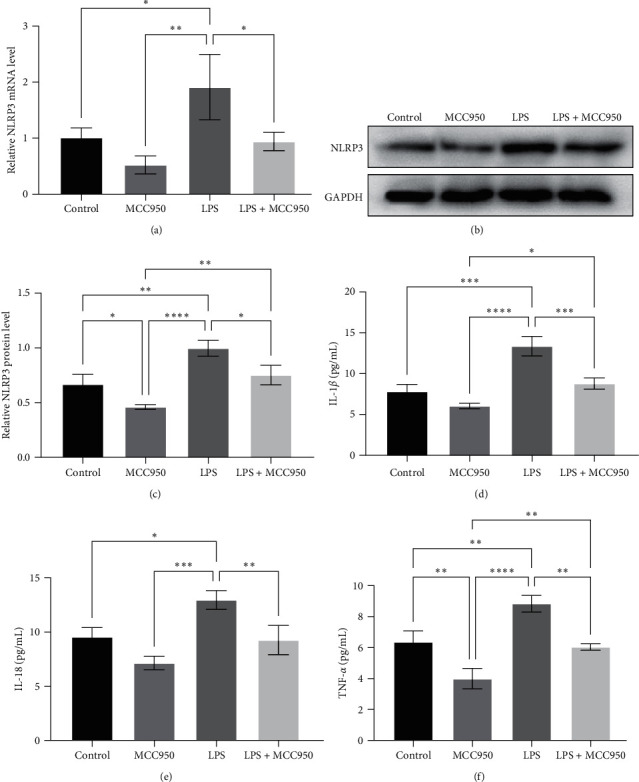
Lipopolysaccharide (LPS) upregulated NLRP3 and inflammatory cytokine expression in bladder cancer cells. In total, 5,637 cells were grown and treated with LPS and/or MCC950 and then subjected to (a) RT-qPCR analysis of NLRP3 mRNA and (b and c) Western blot analysis of NLRP3 protein. The cell culture supernatant was gathered and subjected to enzyme-linked immunosorbent assays (ELISAs) to determine the levels of (d) IL-1*β*, (e) IL-18, and (f) TNF-*α*.  ^*∗*^*P*  < 0.05,  ^*∗∗*^*P*  < 0.01,  ^*∗∗∗*^*P*  < 0.001, and  ^*∗∗∗∗*^*P*  < 0.0001.

Next, we incubated 5,637 cells with 100 *µ*g/mL LPS for 12 hr *in vitro* to mimic the inflammatory tumor microenvironment and assessed NLRP3 expression. Our results revealed that both NLRP3 mRNA and protein were significantly upregulated by LPS treatment (Figure 2(a)–2(c)). The selective small-molecule NLRP3 inhibitor MCC950 (1 *μ*M) was able to significantly downregulate NLRP3 protein expression, even after LPS stimulation (Figures 2(b) and 2(c)) and could also efficiently inhibit the LPS-induced overexpression of NLRP3 mRNA (Figure 2(a)).

Furthermore, we found that LPS was able to markedly increase the levels of IL-1*β*, IL-18, and TNF-*α* expression in 5,637 cells *in vitro* (Figure 2(d)–2(f)). However, MCC950 was able to significantly inhibit the promoting effect of LPS on the expression of these inflammatory factors (Figure 2(d)–2(f)).

### 3.3. LPS Promoted BC Malignant Behaviors through NLRP3 Expression

**Figure 3:**
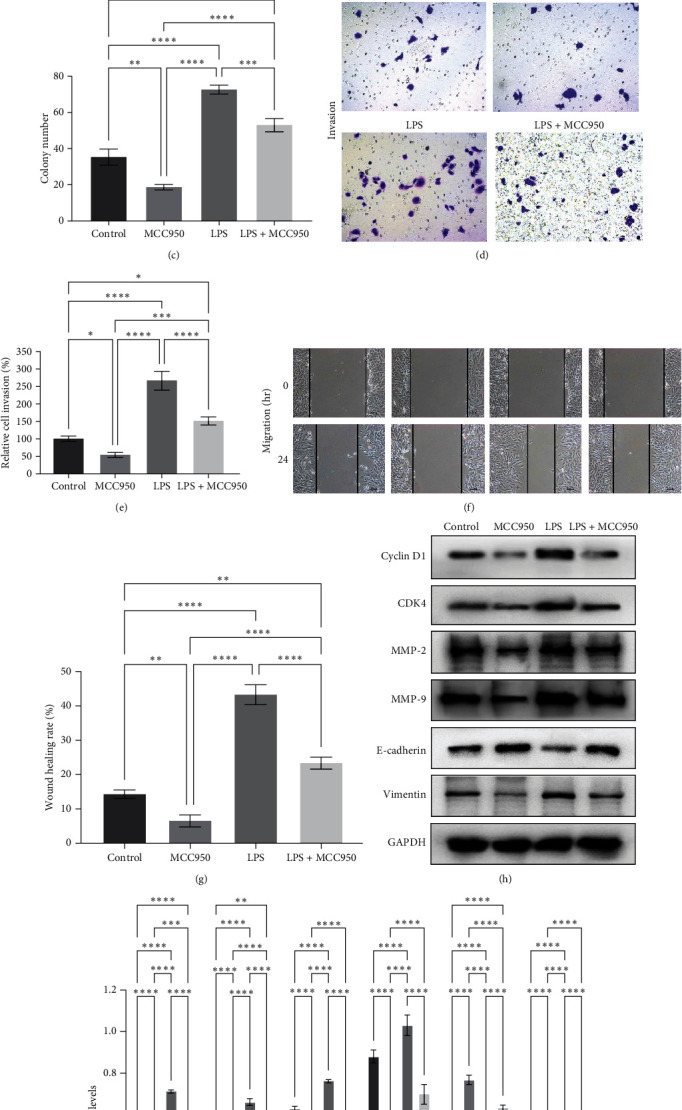
Lipopolysaccharide (LPS) promoted bladder cancer cell malignant behaviors through induction of NLRP3 expression. In total, 5,637 cells were grown and treated with LPS and/or MCC950 and then subjected to the (a) CCK-8 assay, (b and c) colony formation assay, (d and e) transwell invasion assay, (f and g) wound healing assay, and (h and i) Western blot analysis of cyclin D1, CDK4, MMP-2, MMP-9, E-cadherin, and vimentin proteins.  ^*∗*^*P*  < 0.05,  ^*∗∗*^*P*  < 0.01,  ^*∗∗∗*^*P*  < 0.001, and  ^*∗∗∗∗*^*P*  < 0.0001.

After that, we performed CCK-8, colony formation, Transwell invasion, and wound healing assays to assess the effects of the above treatments in BC cells and found that LPS significantly induced BC 5,637 cell viability (Figure 3(a)–3(c)), invasion (Figures 3(d) and 3(e)), and migration (Figures 3(f) and 3(g)) vs. the control cells. However, MCC950 not only decreased the BC cell viability (Figure 3(a)–3(c)), invasion (Figures 3(d) and 3(e)), and migration (Figures 3(f) and 3(g)), but it also significantly alleviated the tumor-promoting effect of LPS on 5,637 cells (Figure 3(a)–3(g)).

Moreover, the Western blot data revealed that LPS was able to significantly upregulate the expression of cyclin D1, CDK4, MMP-2, MMP-9, and vimentin but downregulate the expression of E-cadherin, whereas MCC950 treatment significantly downregulated the expression of cyclin D1, CDK4, MMP-2, MMP-9, and vimentin but upregulated the expression of E-cadherin as well as antagonized the effect of LPS on the expression of these proteins in 5,637 cells (Figures 3(h) and 3(i)).

### 3.4. Cryptotanshinone Inhibited LPS-Induced NLRP3 and Inflammatory Cytokine Overexpression in BC Cells

**Figure 4:**
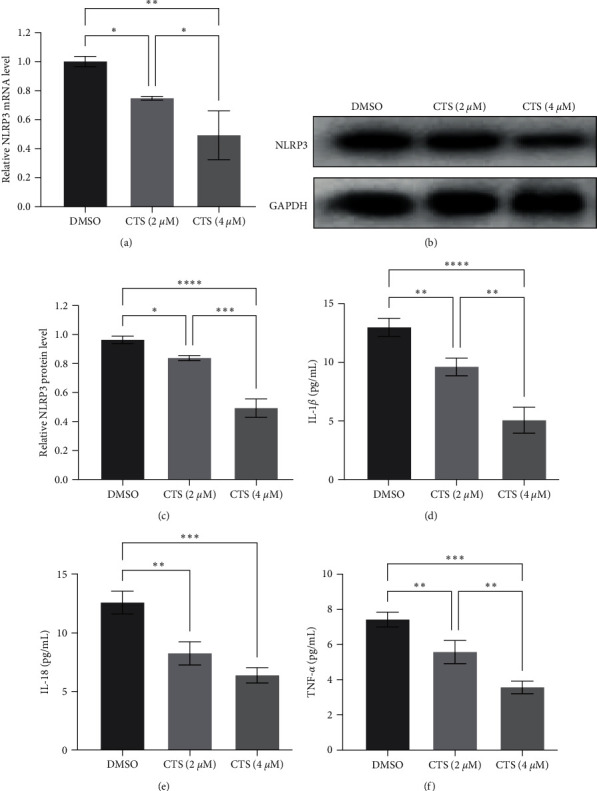
Cryptotanshinone (CTS) dose-dependently inhibited lipopolysaccharide (LPS)-induced NLRP3 and inflammatory cytokine overexpression in bladder cancer cells. In total, 5,637 cells were grown and pretreated with LPS, then treated with CTS, and finally subjected to (a) RT-qPCR analysis of NLRP3 mRNA and (b and c) Western blot analysis of NLRP3 protein. The cell culture supernatant was gathered and subjected to enzyme-linked immunosorbent assays (ELISAs) to determine the levels of (d) IL-1*β*, (e) IL-18, and (f) TNF-*α*. DMSO, dimethyl sulfoxide.  ^*∗*^*P* < 0.05,  ^*∗∗*^*P* < 0.01,  ^*∗∗∗*^*P* < 0.001, and  ^*∗∗∗∗*^*P* < 0.0001.

It is well-known that cryptotanshinone possesses anti-inflammatory, antiproliferative, anti-infective, and antitumor activities in the human body [22, 23]; thus, in this study, we treated 5,637 cells with LPS and then with 2 or 4 *μ*M cryptotanshinone to analyze the changes in NLRP3 and inflammatory cytokine expression. Our results demonstrated that cryptotanshinone markedly decreased the expression of NLRP3 mRNA and protein in 5,637 cells in a dose-dependent fashion (Figure 4(a)–4(c)).

Similarly, cryptotanshinone also inhibited the expression of IL-1*β*, IL-18, and TNF-*α* in 5,637 cells, especially with a significant dose-dependent effect on IL-1*β* and TNF-*α* (Figure 4(d)–4(f)).

### 3.5. Cryptotanshinone Inhibited LPS-Induced BC Cell Malignant Behaviors

**Figure 5:**
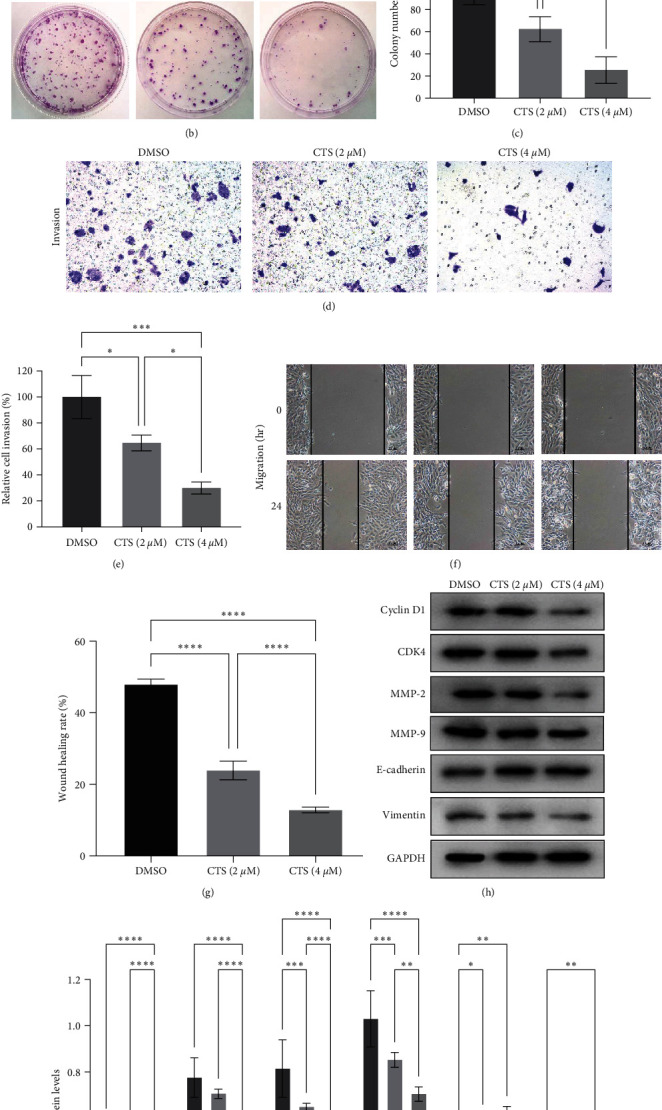
Cryptotanshinone (CTS) inhibited lipopolysaccharide (LPS)-induced bladder cancer cell malignant behaviors in a dose-dependent manner. In total, 5,637 cells were grown and pretreated with LPS, then treated with CTS, and finally subjected to the (a) CCK-8 assay, (b and c) colony formation assay, (d and e) transwell invasion assay, (f and g) wound healing assay, and (h and i) Western blot analysis of cyclin D1, CDK4, MMP-2, MMP-9, E-cadherin, and vimentin proteins. DMSO, dimethyl sulfoxide.  ^*∗*^*P*  < 0.05,  ^*∗∗*^*P*  < 0.01,  ^*∗∗∗*^*P*  < 0.001, and  ^*∗∗∗∗*^*P*  < 0.0001.

We also treated 5,637 cells with LPS and then with 2 or 4 *μ*M cryptotanshinone to analyze the changes in tumor cell malignant behaviors, like cell viability, invasion, and migration. Our data showed that cryptotanshinone reduced the tumor cell viability, invasion, and migration rates in a dose-dependent fashion (Figure 5(a)–5(g)). In addition, cryptotanshinone downregulated the expression of cyclin D1, CDK4, MMP-2, MMP-9, and vimentin as well, but upregulated the E-cadherin level (Figure 5(h)–5(i)).

### 3.6. NLRP3 Overexpression Reversed Cryptotanshinone-Reduced LPS-Induced BC Cell Malignant Behaviors

**Figure 6:**
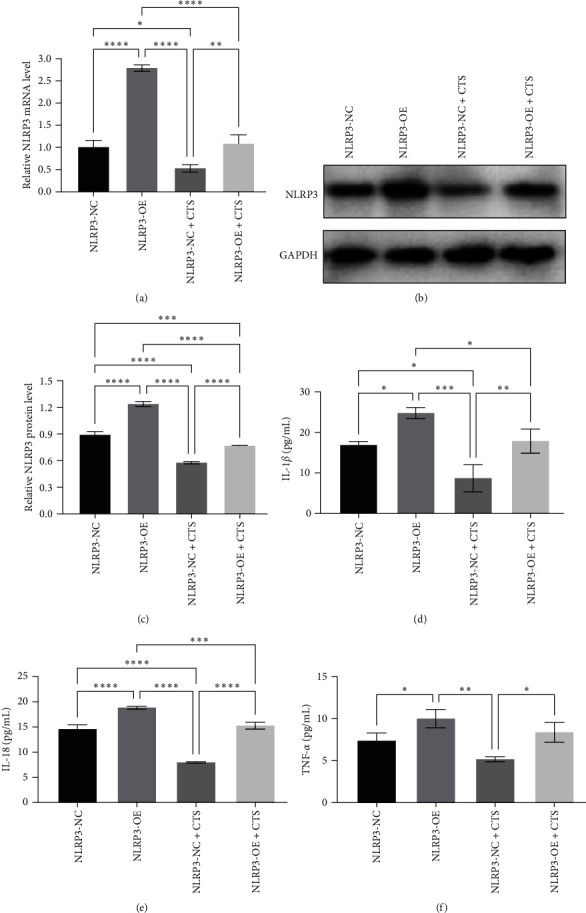
NLRP3 overexpression reversed cryptotanshinone (CTS)-reduced lipopolysaccharide (LPS)-induced inflammatory cytokine expressions in bladder cancer cells. In total, 5,637 cells were grown and pretreated with LPS, then transfected with pcDNA3.1-NLRP3 or vector-only as a negative control, and finally treated with CTS before being subjected to (a) RT-qPCR analysis of NLRP3 mRNA and (b and c) Western blot analysis of NLRP3 protein. The cell culture supernatant was gathered and subjected to enzyme-linked immunosorbent assays (ELISAs) to determine the levels of (d) IL-1*β*, (e) IL-18, and (f) TNF-*α*. NC, negative control; OE, overexpression.  ^*∗*^*P* < 0.05,  ^*∗∗*^*P* < 0.01,  ^*∗∗∗*^*P* < 0.001, and  ^*∗∗∗∗*^*P* < 0.0001.

To confirm the role of NLRP3 on the effects of cryptotanshinone in 5,637 cells, we first pretreated tumor cells with LPS, then transfected them with an NLRP3-overexpression plasmid (pcDNA3.1-NLRP3) and afterward treated them with 4 *µ*M cryptotanshinone. We found that pcDNA3.1-NLRP3 further induced overexpression of NLRP3 mRNA and protein (Figure 6(a)–6(c)) as well as the levels of IL-1*β*, IL-18, and TNF-*α* after LPS treatment (Figure 6(d)–6(f)). Furthermore, the inhibitory effects of cryptotanshinone on NLRP3 and the three inflammatory cytokines were efficiently reversed by NLRP3 overexpression (Figure 6(a)–6(f)).

**Figure 7:**
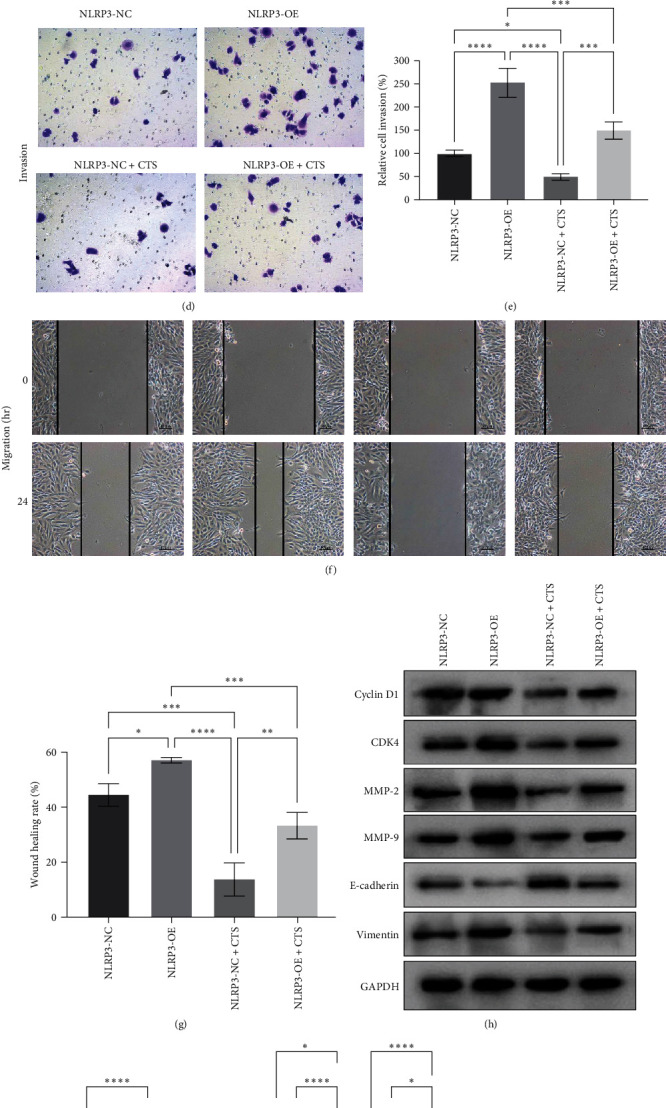
NLRP3 overexpression reversed cryptotanshinone (CTS)-reduced lipopolysaccharide (LPS)-induced bladder cancer cell malignant behaviors. In total, 5,637 cells were grown and pretreated with LPS, then transfected with pcDNA3.1-NLRP3 or vector-only as a negative control, and finally treated with CTS before being subjected to the (a) CCK-8 assay, (b and c) colony formation assay, (d and e) transwell invasion assay, (f and g) wound healing assay, and (h and i) Western blot analysis of cyclin D1, CDK4, MMP-2, MMP-9, E-cadherin, and vimentin proteins. NC, negative control; OE, overexpression.  ^*∗*^*P*  < 0.05,  ^*∗∗*^*P*  < 0.01,  ^*∗∗∗*^ *P*  < 0.001, and  ^*∗∗∗∗*^*P*  < 0.0001.

Meanwhile, we found that NLRP3 overexpression also promoted the tumor cell viability (Figure 7(a)–7(c)), invasion (Figures 7(d) and 7(e)), and migration (Figures 7(f) and 7(g)), as well as the expression of cyclin D1, CDK4, MMP-2, MMP-9, and vimentin but downregulated E-cadherin expression (Figures 7(h) and 7(i)) after LPS treatment. Moreover, NLRP3 overexpression obviously reversed the inhibitory effects of cryptotanshinone on the tumor cell viability, invasion, and migration capacities as well as on expression of these proteins (Figure 7(a)–7(i)).

## 4. Discussion

Our current study assessed NLRP3 expression in BC tissue specimens for association with tumor progression and then explored the inflammatory microenvironment in BC progression using LPS stimulation. After that, we assessed the effects of cryptotanshinone on changes in BC cell malignant behaviors and gene expression. Finally, we confirmed NLRP3 expression in BC progression by using NLRP3 cDNA. Our data exhibited that NLRP3 was likely associated with BC progression. LPS pretreatment significantly induced BC cell viability, migration, and invasion as well as NLRP3 and related inflammatory cytokine expression. In contrast, treatment with cryptotanshinone reduced LPS-induced BC cell malignant behaviors as well as NLRP3 and inflammatory cytokine expression; however, NLRP3 overexpression reversed the inhibitory effects of cryptotanshinone on LPS-induced BC cell malignant phenotypes and the expression of different proteins. Thus, our current study demonstrated that NLRP3 might be an oncogene or at least possess oncogenic activity in BC and that the inhibition of NLRP3 expression by cryptotanshinone might be one of the mechanisms explaining the antitumor activity of cryptotanshinone in BC.

The inflammatory response is a host defense mechanism mediated by immune cells against pathogens and other stimuli [24], and a moderate level of inflammation can protect cells or the body from infection or injury caused by injurious stimuli. However, excessive or sustained inflammatory responses will lead to an imbalance of the immune microenvironment, thus causing harm to the host, even leading to carcinogenesis [25]. To date, long-term infection of hepatitis B or C is believed to cause hepatocellular carcinoma, while human papillomavirus infection is associated with the development of cervical cancer, *Helicobacter pylori* infection is associated with gastric cancer, and colitis is associated with colorectal cancer [25]. Chronic inflammation caused by other conditions or situations, like obesity, tobacco smoking, stress, and an insufficient diet, can also facilitate the occurrence of malignant tumors [26–28]. Moreover, inflammation also has been identified as a critical component of tumor progression [27]. Furthermore, it is widely acknowledged and accepted that uncontrollable or persistent inflammation promotes carcinogenesis and cancer progression through activation of a series of inflammatory signaling pathways [29]. Therefore, improving tumor immunity by regulating inflammatory mediators in the tumor microenvironment is expected to increase the efficacy of tumor therapy in the future.

NLRP3 protein recognizes pathogen-associated molecular patterns as a part of the innate immune system and provides protection of the human body against *Streptococcus pneumonia* infection [30]. Stable activity of NLRP3 is essential for maintaining cellular homeostasis and a healthy body, while abnormal activation of NLRP3 may result in inflammation-related and neoplastic diseases [31] as well as accelerate the progression of malignant tumors [32, 33]; besides, *NLRP3* gene mutations have been correlated to dominantly inherited autoinflammatory diseases like cryopyrin-associated periodic syndrome [34]. A previous pan-cancer study has analyzed NLRP3-inflammasome-related genes in 24 human cancers and has revealed that 15 of 24 cancer types have differential expression of NLRP3-inflammasome-related genes in tumor samples vs. normal ones, and the *NLRP3* gene was generally hypomethylated in 10 of 15 cancer types [35]. In addition, NLRP3 has been reported to be aberrantly upregulated in various tumor tissues, especially in the highly invasive subtype [18], indicating that the NLRP3 inflammasome may be associated with cancer progression, prognosis, and response to treatment [36, 37]. Nevertheless, other evidence has indicated that NLRP3 possesses a key role in antitumor activity through the regulation of host immunity [8, 35]. Thus, the NLRP3 inflammasome may have a bifunctional effect in cancer. Our current study revealed that a high NLRP3 protein level was associated with larger tumor diameters (>2 cm), muscle invasion, and metastasis. Additionally, NLRP3 mRNA and protein were greatly expressed in BC vs. normal urothelial cells. Further studies will confirm the true activity of NLRP3 protein in BC.

LPS, an endotoxin derived from Gram-negative bacteria, has been frequently used to induce experimental fever [38] or other experimental conditions [39–41]. In the current study, we treated BC 5,637 cells with LPS to mimic the inflammatory microenvironment caused by infection and found that LPS notably increased the levels of NLRP3 and inflammatory cytokines, including IL-1*β*, IL-18, and TNF-*α*. LPS also induced BC cell viability, migration, and invasion as well as induced the expression of cell proliferation—and epithelial–mesenchymal transition-related genes, suggesting that the mimetic inflammatory tumor microenvironment may enhance BC malignant behaviors. Furthermore, we transfected NLRP3 cDNA to induce BC cells to overexpress NLRP3 protein, which not only further increased the levels of IL-1*β*, IL-18, and TNF-*α* but also promoted BC cell malignant behaviors and related protein expression, indicating a pivotal role of NLRP3 in the inflammatory microenvironment. It is worth noting that the selective NLRP3 inhibitor MCC950 could exert anticancer activity by suppression of LPS-induced 5,637 cell malignant behaviors. Therefore, our current study supports that NLRP3 is considered as an oncogene in BC and that targeting NLRP3 expression or activity could be a novel BC therapeutic strategy in the future.

Cryptotanshinone, a natural antibacterial agent, possesses different bioactivities, including *anti-inflammatory*, *antiproliferative*, and *anti-infective* activities [22]. It has been reported that cryptotanshinone possesses anticancer activity by inhibiting the signal transduction and activator of transcription-3 in prostate cancer [42]. Additionally, a review article has summarized the potential use of cryptotanshinone for cancer immunotherapy and as a sensitizer of tumor cells to chemotherapeutic agents [21]. In BC, a recent study has revealed that cryptotanshinone was able to inhibit tumor cell proliferation and induce the cells to undergo apoptosis; molecularly, cryptotanshinone activated phosphatase and tensin homolog to suppress the phosphatidylinositol 3 kinase/protein kinase B signaling pathway [43]. Meanwhile, another recent report has shown that cryptotanshinone, as a novel pyruvate dehydrogenase kinase 4 inhibitor, reduced the BC cell invasion capacity by regulating the mammalian target of rapamycin/*β*-catenin/N-cadherin signaling pathway [44]. To date, there are only three published studies of cryptotanshinone in BC [43–45]. Our current study added more data on cryptotanshinone in BC *by showing that this compound inhibited* NLRP3 expression and reduced BC cell malignant phenotypes *in vitro* with LPS pretreatment. Our current findings are consistent with these three previous studies [43–45]. To confirm the effect of *cryptotanshinone on* NLRP3 inhibition, we also performed an NLRP3 rescue experiment and found that NLRP3 overexpression significantly reversed the inhibitory activity of *cryptotanshinone against* LPS-induced 5,637 cell malignant phenotypes and gene expression.

However, this study also has some limitations that must be addressed. For example, the number of BC tissue samples assessed in this study was not large enough, and the patients were not followed up for association studies of NLRP3 expression with survival data. Besides, the simulated inflammatory microenvironment using LPS may be different from the inflammatory microenvironment *in vivo*. Moreover, it is unclear whether *cryptotanshinone* directly or indirectly targets NLRP3 expression, and further studies are needed. Overall, this study is just a proof-of-principle study and further investigation will clarify the intrinsic mechanisms.

## 5. Conclusions

In conclusion, high NLRP3 expression is likely to be a risk factor for BC progression, and the LPS-induced inflammatory microenvironment can promote NLRP3 overexpression and BC malignant behaviors *in vitro*. Cryptotanshinone treatment was able to inhibit cancer cell progression, probably through inhibiting NLRP3. Future studies are needed to confirm the antitumor activity of cryptotanshinone in BC in the clinic.

## Figures and Tables

**Table 1:** Association of NLRP3 levels with clinicopathological features from 62 bladder cancer patients.

Variables	*N*	Strong expression *n* (%)	Moderate expression *n* (%)	Weak/negative expression *n* (%)	*P*-value
Sex					
Male	45	12 (26.67)	15 (33.33)	18 (40.00)	0.753
Female	17	5 (29.41)	4 (23.53)	8 (47.06)	—
Age (years)					
<68	31	9 (29.04)	11 (35.48)	11 (35.48)	0.563
≥68	31	8 (25.81)	8 (25.81)	15 (48.38)	—
Tumorigenesis frequency					
Primary	44	10 (22.73)	13 (29.54)	21 (47.73)	0.288
Recurrence	18	7 (38.89)	6 (33.33)	5 (27.78)	—
Tumor number					
Unicity	28	7 (25.00)	10 (35.71)	11 (39.29)	0.733
Multiplicity	34	10 (29.41)	9 (26.47)	15 (44.12)	—
Tumor diameter					
≤2 cm	33	5 (15.15)	10 (30.30)	18 (54.55)	0.038 ^*∗*^
>2 cm	29	12 (41.38)	9 (31.03)	8 (27.59)	—
Invasive type					
NMIBC	38	6 (15.79)	14 (36.84)	18 (47.37)	0.034 ^*∗*^
MIBC	24	11 (45.84)	5 (20.83)	8 (33.33)	—
Metastasis					
Yes	17	9 (52.94)	5 (29.41)	3 (17.65)	0.012 ^*∗*^
No	45	8 (17.78)	14 (31.11)	23 (51.11)	—
Histological grade					
High	36	11 (30.56)	10 (27.78)	15 (41.66)	0.764
Low	26	6 (23.08)	9 (34.61)	11 (42.31)	—
Smoking					
Yes	27	7 (25.93)	8 (29.63)	12 (44.44)	0.939
No	35	10 (28.57)	11 (31.43)	14 (40.00)	—
Drinking					
Yes	25	4 (16.00)	8 (32.00)	13 (52.00)	0.220
No	37	13 (35.14)	11 (29.72)	13 (35.14)	—
Hypertension					
Yes	33	11 (33.33)	10 (30.30)	12 (36.37)	0.490
No	29	6 (20.69)	9 (31.03)	14 (48.28)	—
Diabetes					
Yes	15	6 (40.00)	5 (33.33)	4 (26.67)	0.318
No	47	11 (23.40)	14 (29.79)	22 (46.81)	—
Body mass index (kg/m^2^)					
<24	31	8 (25.80)	10 (32.26)	13 (41.94)	0.946
≥24	31	9 (29.03)	9 (29.03)	13 (41.94)	—

NMIBC, nonmuscle-invasive bladder cancer; MIBC, muscle-invasive bladder cancer.  ^*∗*^*P*  < 0.05.

## Data Availability

The data underlying this article will be shared upon reasonable request to the corresponding author.
